# Histopathologic indicators of breast cancer biology: insights from population mammographic screening

**DOI:** 10.1038/sj.bjc.6602501

**Published:** 2005-04-05

**Authors:** L R Webster, A M Bilous, L Willis, K Byth, F C Burgemeister, E L C Salisbury, C L Clarke, R L Balleine

**Affiliations:** 1 Translational Oncology Westmead and Nepean Hospitals, Westmead, NSW 2145, Australia; 2 Westmead Institute for Cancer Research, Westmead Millennium Institute, Westmead, NSW 2145, Australia; 3 University of Sydney, Camperdown, NSW 2006, Australia; 4 Department of Tissue Pathology, Institute of Clinical Pathology and Medical Research Westmead Hospital, Westmead, NSW 2145, Australia; 5 BreastScreen Greater Western Sydney, Parramatta, NSW 2150, Australia; 6 Division of Medicine, Westmead Hospital, Westmead, NSW 2145, Australia

**Keywords:** breast cancer, histopathology, mammographic screening

## Abstract

Histopathologic features of breast cancer such as tumour size, grade and axillary lymph node (LN) status variably reflect tumour biology and time. Recent evidence suggests that the biological character of breast cancer is established at an early stage and has a major impact on clinical course. The aim of this study was to distinguish the impact of biology on breast cancer histopathology by comparing features of breast cancers diagnosed following population mammographic screening with prevalent *vs* incident detection and screening interval. Central histopathology review data from 1147 cases of ductal *in situ* and/or invasive breast cancer were examined. Size, grade and LN status of invasive cancers were positively correlated (*P*<0.001). Prevalent invasive cancers were larger (*P*<0.001) and more likely to be LN positive (*P*=0.02) than incident cases, but grade was not associated with screening episode (*P*=0.7). Screening interval for incident cancers was positively associated with invasive cancer size (*P*=0.05) and LN status (*P*=0.002) but not grade (*P*=0.1). Together, these data indicate that biology and time both impact on size and LN status of invasive breast cancer, but grade reflects biology alone. In view of the clinical importance of breast cancer biology, grade as its most direct indicator assumes particular significance.

Histopathologic assessment of breast cancer has long provided the basis for prediction of recurrence risk and prescription of adjuvant therapy. The features routinely documented include tumour size, type, grade and the presence of axillary lymph node (LN) metastases (Australian Cancer Network, 2001). In addition, presence of oestrogen and progesterone receptors (ER*α* and PR) is assessed principally to indicate the potential value of endocrine therapy (Elledge *et al*, 2000).

Recently, gene expression studies, which combine data on expression of thousands of genes with powerful computational analysis, have given new insight into breast cancer biology. In particular, these studies have demonstrated that subtypes of breast cancer with a predictable clinical course can be defined and reproducibly identified on the basis of gene expression patterns (Sorlie *et al*, 2001, 2003; van de Vijver *et al*, 2002; van't Veer *et al*, 2002). In this is a compelling demonstration that tumour biology has a pervasive impact on clinical outcome. Furthermore, evidence that the essential biological character of breast cancer is apparent early in disease development comes from the detection of the same discriminant gene expression profiles in early-stage breast cancer, more advanced lesions and metastases (van de Vijver *et al*, 2002; Ma *et al*, 2003; Ranaswamy *et al*, 2003).

In common with global gene expression patterns, the histopathologic features of breast cancer reflect the influence of a number of biological processes including not only those operating in cancer cells but also cancer–stromal interactions and complex immune and hormonal influences. However, in addition to biology, the histopathology ‘snapshot’ of a cancer incorporates the period of opportunity that morphological features have had to develop. In the light of recent revelations on the natural history and clinical importance of breast cancer biology, it is timely to consider how biology is reflected in histopathologic parameters.

Population mammographic screening, effecting preclinical detection and regular re-examination, provides an opportunity to examine the relative contribution of biology and time to development of the major histopathologic features of breast cancer. We have examined this issue in a large cohort of screen detected breast cancers that were subject to central histopathology review. Pathologic features of breast cancer diagnosed at a first screening round (prevalent) were compared with incident cases on the basis that prevalent cancers have potentially longer to develop prior to detection than incident cancers where this is theoretically limited by screening interval. In addition, for incident invasive breast cancers, histopathologic features were compared with the interval between the diagnostic screening round and the previous negative screen.

## PATIENTS AND METHODS

### Patient cohort

Patients were women diagnosed with breast cancer, as a consequence of mammographic screening, by the *BreastScreen Greater Western Sydney* unit in New South Wales Australia, in the period from the commencement of the screening programme in 1993 to February 2000. In this period, 1519 women were diagnosed with invasive breast cancer or ductal carcinoma *in situ* (DCIS) that was subsequently confirmed in an excision specimen. At the time of analysis, cancers from 1159 of these individuals had been subject to central histopathology review. This group forms the study cohort following further exclusion of 11 patients with recurrent breast cancer, six with interval cancer and three with review data unavailable. In the final study cohort, cases of simultaneously diagnosed bilateral breast cancer were included as two separate cancers (*n*=8). In patients with multiple foci of cancer in the same breast (*n*=43), only features of the largest lesion were included. Hence, data from 1147 separate cancers in 1139 patients were analysed: 977 invasive cancers (including 572 with coexisting DCIS) and 170 cases of DCIS only.

Information on patient age and screening round of diagnosis was derived from the records of *BreastScreen Greater Western Sydne*y. In general, following an initial screen, women are invited to attend for rescreening at intervals of 21–27 months (2±0.25 years), except that annual screens are offered to individuals with a clinical indicator of increased breast cancer risk detected at screening, or who have a first-degree relative with a history of breast cancer. Individuals who choose not to attend for rescreening at invitation may present for rescreening at any subsequent time. Any mammograms taken for screening or other purposes outside the operations of the *BreastScreen Greater Western Sydne*y screening unit were not taken account of in this analysis.

Cancers diagnosed at the initial screening episode were designated *prevalent.* Cancers diagnosed at a round subsequent to one or more screens where cancer was not detected were *incident*.

### Pathology review

Central pathology review was conducted at the Institute for Clinical Pathology and Medical Research (ICPMR), Westmead Hospital. Original pathology reports and slides from a number of regional laboratories were examined by senior pathologist trainees under the supervision of a single experienced breast pathologist (AMB). Histopathological features were documented according to a standardised format and included: invasive cancer size, type, grade (Elston and Ellis, 1991), and the number and status of excised axillary LNs. In 479 of 742 (65%) cases that were DCIS only or DCIS associated with invasive cancer, one or more DCIS grades was assigned according to the options: high grade (comedo), intermediate grade, low grade (noncomedo) or mixed nuclear grades.

This project received institutional Human Research Ethics Committee approval.

### Statistical analysis

Statistical analyses were performed using SPSS for Windows Version 12.0 (SPSS Science, Chicago, US). Tumour size, LN status and screening interval were categorised in most analyses as shown in relevant figures and tables referred to in the text. Tumour size and screening interval were compared both as continuous and categorical variables. Pearson's *χ*^2^ test (or Fisher's exact test if an expected cell size <5) was used to test for association between categorical variables. Spearman's rank correlation (*r*) was used to quantify the degree of association between ordered categorical variables or continuous variables. Multiple logistic regression analysis was used to test for interaction between the effects of episode type and histopathological grade on LN positivity. A general linear model was used to test for interaction between the effects of grade and episode type on tumour size. *P*-values ⩽0.05 were considered to be statistically significant.

## RESULTS

### Patient and tumour characteristics

Patient and tumour characteristics are summarised in Table 1.

In this cohort, accrued from the commencement of a population mammographic screening programme, 699 (61%) cancers were prevalent and 446 (39%) incident. The majority of incident cancers were diagnosed at screening rounds 2 (*n*=256, 57.4%) and 3 (*n*=152, 34.1%), with the remaining small proportion diagnosed at rounds 4–6 (*n*=38, 8.5%). The age at diagnosis ranged from 40.1 to 92.6 years (median 59.9). This was similar to the age at first screen for the cohort (40.1–92.6, median 58.7 years), reflecting the high proportion of cases diagnosed at the first screening round.

The predominant histological type of invasive cancers was *ductal not otherwise specified* (83.3%). The overall distribution of invasive cancer grades was 39.9% grade 1, 39% grade 2 and 21.1% grade 3. The majority of invasive cancers were of relatively small size with 37.8% ⩽10 mm in maximum dimension and 83.4% ⩽20 mm. Most invasive cancers were axillary LN negative (72.5%).

There were 170 reviewed cases consisting entirely of DCIS. In 105 of these graded according to the schema outlined in Table 1, 57.1% were designated *high grade (comedo)* or a combination of grades including *high grade (comedo).*

### Inter-relationship of histopathological features of invasive cancer

**Figure 1:**
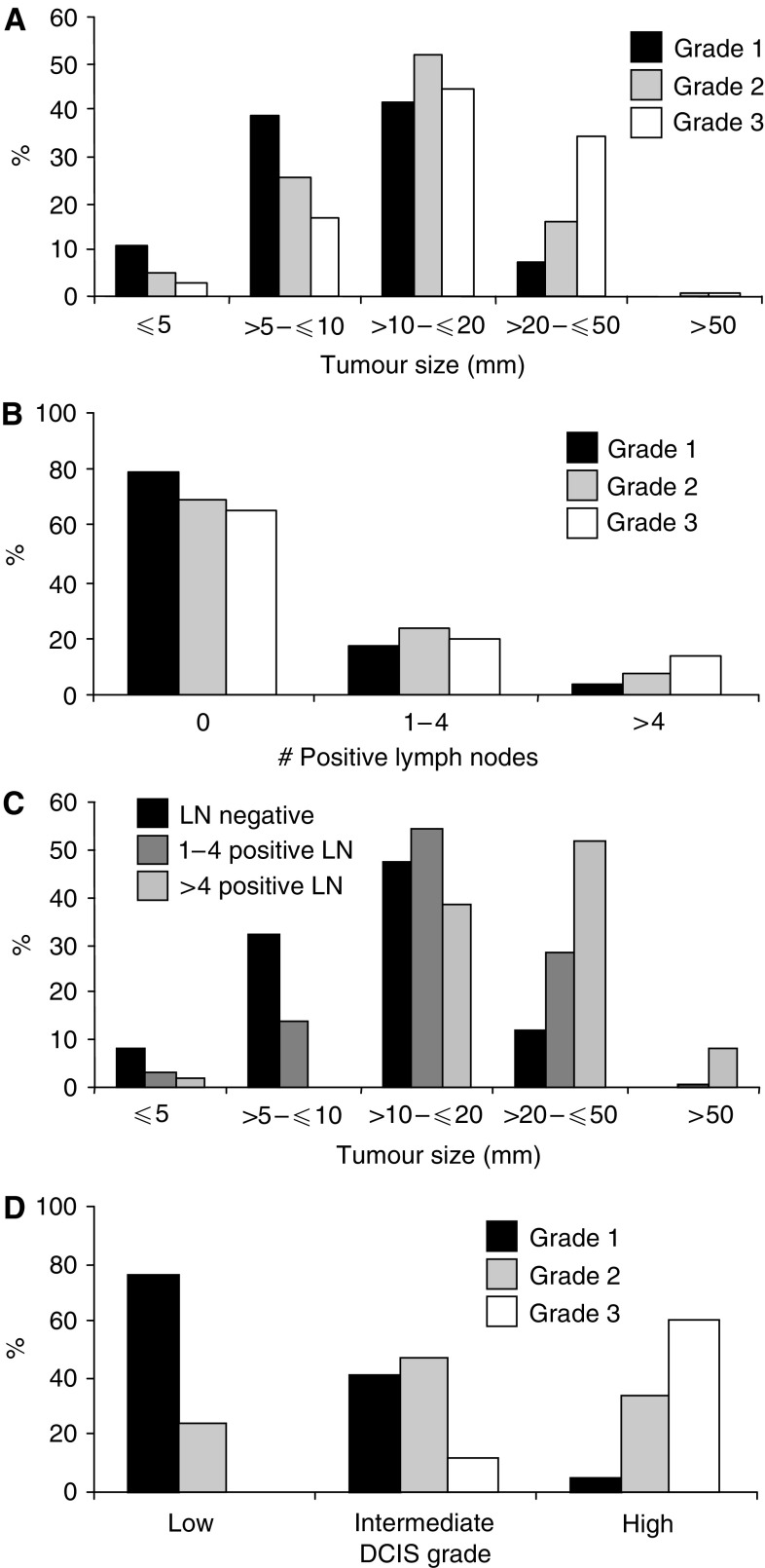
(**A–D**) Inter-relationship of histopathologic features of invasive breast cancer: (**A**) Grade *vs* tumour size *n*=921 (*y*-axis: % of each grade category), *P*<0.001, *r*=0.31. (**B**) Grade *vs* LN status *n*=810 (*y*-axis: % of each grade category), *P*<0.001, *r*=0.14. (**C**) LN status *vs* tumour size *n*=811 (*y*-axis: % of each LN category), *P*<0.001, *r*=0.35. (**D**) Invasive cancer grade *vs* DCIS grade. Only cases with DCIS grade designated solely as low grade (noncomedo), intermediate grade or high grade (comedo) were included in this analysis *n*=273 (*y*-axis: % of each invasive cancer grade category), *P*<0.001, *r*=0.7. Abbreviations: LN, lymph node.

The histopathological features of invasive breast cancer were positively correlated such that higher grade was associated with larger tumour size (*P*=0.001, *r*=0.31, Figure 1A) and axillary LN metastases (*P*<0.001, *r*=0.14, Figure 1B). Larger tumour size was also related to the presence of LN metastases (*P*<0.001, *r*=0.35, Figure 1C). Where DCIS was coexistent with invasive cancer, and a single uniform DCIS grade was recorded, this was significantly correlated with the invasive cancer grade (*P*<0.001, *r*=0.70, *n*=273, Figure 1D).

### Pathological features of invasive cancer and diagnostic screening episode

Prevalent invasive breast cancers were on the whole larger (*P*<0.001) and more commonly LN positive than incident cases (*P*=0.02). In contrast, the distribution of invasive cancer grades was not different between prevalent and incident detected cancers (*P*=0.7, Table 2A).

The association between diagnostic screening episode and invasive cancer size was independent of grade (*P*=0.1). When LN status was considered in relation to both invasive cancer grade and episode type, the demonstrated association between LN positivity and prevalent screening round appeared strongest for lower-grade cancers. However, this distinction was not statistically significant (grade by episode-type interaction for LN status *P*=0.1, Table 2B).

### Screening interval of incident invasive cancer and DCIS

**Figure 2:**
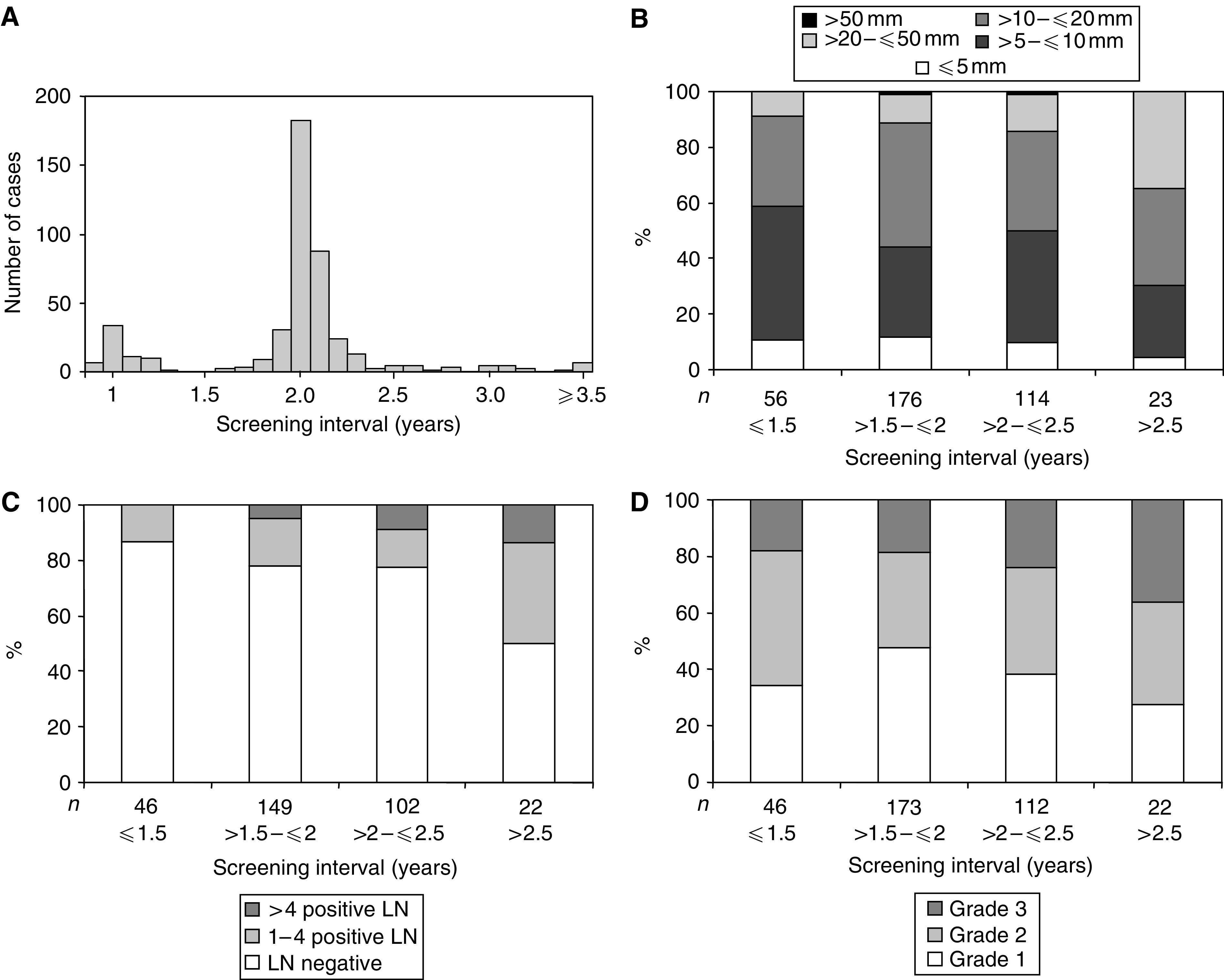
(**A**) Distribution of screening intervals for incident cases *n*=446 (invasive cancer *n*=378, DCIS *n*=68). (**B**) Comparison between screening interval and invasive breast cancer size, represented as a proportion of each screening interval category *n*=369, *P*=0.03. (**C**) Comparison between screening interval and axillary LN status, represented as a proportion of each screening interval category *n*=319, *P*=0.002. (**D**) Comparison between screening interval and invasive cancer grade, represented as a proportion of each screening interval category *n*=362, *P*=0.1. Abbreviations: LN, lymph node.

For incident cases, the interval between the diagnostic screening round and the previous negative screen (screening interval) ranged from 0.9 to 5.4 years with a median interval of 2.0 years. Screening interval for the majority of cases was consistent with the usual recommended screening interval of 2±0.25 years: the 25 to 75 percentile range for screening interval was 2.0–2.1 years and screening interval was equivalent to the median of 2.0 years for over 40% of cases. A screening interval of 1 year or less was recorded for 9% of cases. Conversely, 6% of cases had a screening interval longer than 2.5 years (Figure 2A).

### Pathological features of incident invasive cancer and screening interval

There was a weak but significant positive correlation between screening interval and incident invasive breast cancer size (*P*=0.05, *r*=0.10 for screening interval and size as continuous variables, *P*=0.03 for categories as shown in Figure 2B). The relationship between screening interval and tumour size was independent of grade (*P*=0.9).

The presence and number of axillary LN metastases was also positively associated with screening interval (*P*=0.002, Figure 2C).

There was no statistically significant association between grade and screening interval category (*P*=0.1, Figure 2D).

## DISCUSSION

In this study, influence of the period of development of invasive breast cancer prior to diagnosis was judged by comparing prevalent screen detected cancers with incident cases and also by examining the effect of screening interval on features of incident cases. Clearly, the size of breast cancer increases with time and inferences about time inherent in this analysis are supported by our finding that prevalent cancers were generally larger than incident cases and size of invasive cancers was positively correlated with screening interval. Histopathologic grade was not related to diagnostic screening round or screening interval which, in combination with the correlation between grade of *in situ* and invasive disease, supports the conclusion that grade is not influenced by time but directly reflects cancer biology. Invasive cancer size and LN status were each strongly correlated with grade in addition to being related to diagnostic screening round and screening interval, consistent with a combined influence of biology and time on these features.

**Figure 3:**
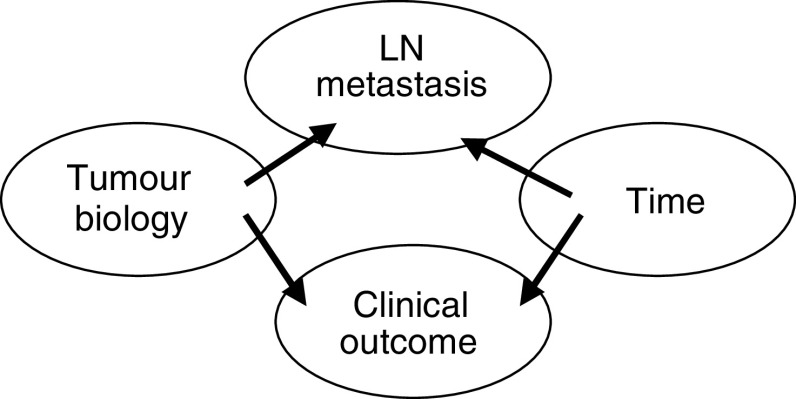
Schematic representation of the disparate influence of time and breast cancer biology on clinical indicators and end points.

These findings give insight into the demonstration that a prognostic gene expression signature in breast cancer correlated strongly with grade, less strongly with tumour size and was not related to axillary LN status (van de Vijver *et al*, 2002), since the impact of biology on these later two features is time dependent. Furthermore, a unifying hypothesis to explain the apparent paradox of failure of a direct relationship between the prognostic gene expression signature and LN status given the well-established prognostic significance of axillary LN metastases (Elston *et al*, 1999; Singletary *et al*, 2002) may be that the end points of LN positivity and clinical outcome are each determined by biology and time, but the impact of time on each of these is variable, dependent on biology and not necessarily equal (Figure 3).

To generate a prognostic gene expression signature, a large number of biological indicators are revealed, and then ranked according to their combined ability to discriminate between cases that relapse and those that do not (van't Veer *et al*, 2002). This is an essentially different process than was used to establish current clinicopathologic prognostic indicators. Interestingly, many of the biological pathways that have emerged as influential in discriminative gene sets, such as hormone receptor activation and cellular proliferation (van't Veer *et al*, 2002; Gruvberger *et al*, 2003), have long been known to be important in distinguishing different types of breast cancer and are routinely assessed. However, the superior prognostic power of gene expression studies suggests that prediction of clinical course using commonly available clinicopathological parameters could be improved. In this regard, it is necessary to discern the influence of biology in histopathological features and the variable influence of time on tumour size and LN status is important to acknowledge.

A potential weakness of histopathologic assessment of breast cancer is the influence of interobserver variability on the features reported. In the presented cohort, this effect is reduced because data were derived from central review of diagnostic material. Another general limitation of breast cancer histopathology reporting is the absence of a robust, universally accepted method for grading DCIS (Consensus Conference Committee, 1997). In the current report, an overall descriptive assessment of DCIS grade was recorded, and notwithstanding the limitations of this approach, a strong correlation between the grade of DCIS and concomitant invasive cancer was found, which is consistent with previous reports (Gupta *et al*, 1997; Leong *et al*, 2001) and data from cytogenetic studies showing distinct patterns of chromosomal aberration in low- and intermediate-grade invasive cancer and DCIS of the same grades (Buerger *et al*, 1999a, 1999b, 2001).

The presented cohort of screen detected breast cancers was accrued from the beginning of a population screening programme and selected on the basis of completed central histopathology review. Despite this, the overall histopathologic features of the cohort are typical of screen detected invasive breast cancer, which has been repeatedly demonstrated to be generally smaller, lower grade and less likely to be LN positive than cancers presenting clinically (Cowan *et al*, 1997; Ernst *et al*, 2002; Vacek *et al*, 2002).

Diagnosis of breast cancer at an early stage is the principal aim of mammographic screening and the relatively small size and low rate of LN positivity in screen detected cancers demonstrates this effect. However, as grade is a time-independent indicator of biology, the overall lower grade of screen detected invasive cancers indicates an additional more complex interaction between screening diagnosis and breast cancer biology. This preferential detection of lower-grade cancers is consistent with the ‘length bias’ inherent in population cancer screening, whereby cancers that progress slowly are preferentially detected as these have a longer preclinical period amenable to screen diagnosis (‘sojourn time’) (Cole and Morrison, 1980; IARC, 2002). Conversely aggressive, rapidly progressing cancers are more likely to reach the threshold for clinical detection before a patient presents for screening or in the interval between routine screens and will therefore be reduced in a screen detected cancer cohort. In our data set, indirect support for an impact of length bias on the distribution of invasive cancer grades comes from DCIS-only cases, which are unlikely to be diagnosed clinically, and which showed a proportion of high-grade cases (57.1%) that was greater than high-grade invasive cancers (21.2%) and more similar to the reported rate of high-grade invasive cancers in a population not selected by detection method (47%) (Elston and Ellis, 1991).

According to population cancer screening theory, ‘length bias’ is greatest at the prevalent screening round and may be almost eliminated by the third screening round if the screening interval is short (Cole and Morrison, 1980). If length bias is eliminated, this theory predicts a greater proportionate representation of aggressive fast-growing cancers among incident cases. However, in our cohort, the overall larger size of prevalent cancers compared with incident cases argues against a loading of rapidly progressing cases in the later group. The apparent persistence of length bias among incident invasive cancers in this cohort may be attributable to the fact that the majority (57.4%) of incident cases were detected at the second screening round. It is also possible that overall the screening interval was not sufficiently short relative to sojourn time of rapidly progressing cancers to eliminate the effect of length bias for the entire cohort.

The advantages of this study compared with previous analyses of screen detected breast cancer pathology are not only the large cohort size and central review format but also data from contemporary research, in particular gene expression studies, that give fresh insight into the results. Rational interpretation of histopathologic features of breast cancer is especially important in the era of population mammographic screening as cancers with disparate biology may similarly present as small and LN negative. In the light of new knowledge about the importance of tumour biology, grade as its most direct histopathologic indicator assumes particular significance.

## Figures and Tables

**Table 1:** Patient and tumour characteristics

	***n* (%)**
All cancers	1147
Invasive cancer	977 (85.2)
Invasive cancer+DCIS	572
DCIS only	170 (14.8)
	
*Episode type (n*=*1145*^a^)	
Prevalent	699 (61.0)
Incident	446 (39.0)
	
*Age at first screen*	
Range (median)	40.1–92.6 (58.7)
	
*Age at diagnosis*	
Range (median)	40.1–92.6 (59.9)
	
*Invasive cancers*	977
Histological type	
Ductal NOS	814 (83.3)
Invasive lobular, classical	47 (4.8)
Tubular carcinoma	35 (3.6)
Mucinous carcinoma	29 (3.0)
Invasive lobular, variant	20 (2.0)
Other/mixed	32 (3.3)
	
*Grade (n*=*944*^b^)	
1	377 (39.9)
2	368 (39.0)
3	199 (21.1)
	
*Tumour size (mm) (n*=*949*^c^)	
⩽5	83 (8.8)
>5–⩽10	275 (29.0)
>10–⩽20	433 (45.6)
>20–⩽50	152 (16.0)
>50	6 (0.6)
	
*LN positive (n*=*829*^d^)	
0	601 (72.5)
1–4	166 (20.0)
>4	62 (7.5)
	
*DCIS*+*invasive cancer*	
Histological type^e^	374^f^
Low grade (noncomedo)	81 (21.7)
Intermediate grade	135 (36.1)
High grade (comedo)	129 (34.5)
Ductal – mixed nuclear grades	29 (7.8)
	
*Pure DCIS*	
Histological type^e^	105^g^
Low grade (noncomedo)	9 (8.5)
Intermediate grade	29 (27.6)
High grade (comedo)	60 (57.1)
Ductal – mixed nuclear grades	7 (6.7)

DCIS=ductal carcinoma *in situ*; LN=lymph nodes; NOS=not otherwise specified.

a Data not available for two cases.

b Tumours too small to grade (*n*=13), grade not recorded (*n*=20).

c Size was not recorded at review (*n*=28).

d LNs were not removed or not reviewed (*n*=148).

e Cases with more than one DCIS histological type recorded are included in the highest grade category: *n*=67 for DCIS with invasive cancer (*n*=56 included as intermediate grade and *n*=11 as high grade) and *n*=23 for the pure DCIS group (*n*=18 included as intermediate grade and *n*=5 as high grade).

f Excludes 198 and 65 cases not graded according to this scheme.

g Excludes 198 and 65 cases not graded according to this scheme.

**Table 2:** Relationship between histopathological features of invasive cancers and diagnostic screening episode. (A) Episode type by tumour size, LN status and grade and (B) proportion LN positive by grade and episode type

	**Prevalent, *n* (%)**	**Incident, *n* (%)**	
*(A)* a			
Tumour size (mm) (*n*=948)			
⩽10	182 (31.4)	175 (47.4)	*P*<0.001
>10–⩽20	288 (49.8)	145 (39.3)	
>20	109 (18.8)	49 (13.3)	
			
LNs (*n*=829)			
Negative	355 (69.6)	246 (77.1)	*P*=0.02
Positive	155 (30.4)	73 (22.9)	
			
Grade (*n*=943)			
1	226 (38.9)	150 (41.4)	*P*=0.7
2	233 (40.1)	135 (37.3)	
3	122 (21.0)	77 (21.3)	
			
*(B)* b			
Grade	**Proportion LN positive**		
1	46/182 (25.3)	16/118 (13.6)	*P*=0.1
2	70/204 (34.3)	32/121 (26.4)	
3	39/113 (34.5)	24/72 (33.3)	

LN=lymph nodes.

a *P*-values from Pearson *χ*^2^ test.

b *P*-value represents grade by episode-type interaction tested using multiple logistic regression analysis of LN positivity.
